# Patient experience with hospital care following the Maryland global budget revenue model: A difference-in-difference analysis

**DOI:** 10.1371/journal.pone.0308331

**Published:** 2024-08-06

**Authors:** Ronnie L. Shammas, Jenna Li, Evan Matros, Oluseyi Aliu

**Affiliations:** 1 Department of Surgery, Division of Plastic, Maxillofacial, and Oral Surgery, Duke University, Durham, NC, United States of America; 2 Allegheny Singer Research Institute, Allegheny Health Network, Pittsburgh, PA, United States of America; 3 Department of Surgery Memorial Sloan Kettering Cancer Center, Plastic and Reconstructive Surgery Service, New York, NY, United States of America; 4 Department of Surgery, Division of Plastic and Hand Surgery, Allegheny Health Network, Pittsburgh, PA, United States of America; Murcia University, Spain, SPAIN

## Abstract

**Introduction:**

As a result of the success of Maryland’s full risk capitated payment model experiment (Global Budget Revenue) in constraining healthcare costs, there is momentum for expanding the reach of such models. However, as these models are implemented, studies analyzing their long-term effects suggest unintended spillover effects that may ultimately influence patient experiences. The aim of this study was to determine whether implementation of the GBR was associated with changes in patient experience.

**Methods:**

Cross-sectional study using a difference-in-difference analysis to examine changes in patient experiences according to the Hospital Consumer Assessment of Healthcare Providers and Systems (HCAHPS) domains before and after implementation of the GBR model. Acute care hospitals from 2010–2016 with completed HCAHPS surveys were included. Hospitals identified for inclusion were then matched, based on county location, to area level characteristics using the Area Health Resource File.

**Results:**

A total of 844 hospitals were included. Compared to hospitals in non-GBR states, hospitals in GBR states experienced significant declines in the following HCAHPS domains: “would definitely recommend the hospital to others” [Average treatment effect (ATT) = -1.19, 95% CI = -1.97, -0.41)] and 9–10 rating of the hospital (ATT = -0.93, 95% CI = -1.71, -0.15). Results also showed significant increases in the HCAHPS domains: “if patient’s rooms and bathroom were always kept clean” (ATT = 1.10, 95% CI = 0.20, 2.00). There were no significant differences in changes for the other domains, including no improvements in: nursing communication, doctor communication, help from hospital staff, pain control, communication on medicines, discharge information, and quietness of the patient environment.

**Conclusion:**

These findings suggest there should be efforts made to ascertain and mitigate potential adverse effects of care transformation initiatives on patient experience. Patients are stakeholders and their inputs should be sought and incorporated in care transformation efforts to ensure that these models align with improved patient experiences.

## Introduction

The state of Maryland’s full risk capitated payment model experiment (Global Budget Revenue) which ran from 2014–2019 demonstrated success in constraining healthcare costs to the tune of almost 1 billion dollars [1, 2]. Additionally, there were improvements in objective patient outcomes (i.e., complications, in-hospital admissions, length of stay, mortality) through the model’s running period [3–9]. Although the Centers for Medicare and Medicaid Services (CMS) through the Center for Medicare and Medicaid Innovation (CMMI) has overseen experiments with other payment reform models (e.g., bundled payments), based on the success of Maryland’s GBR, CMS has signaled a commitment to accelerating the adoption of full risk capitated models. Examples demonstrating this commitment include Maryland’s Total Cost of Care Model (TCOC), Pennsylvania’s Rural Hospital Model and the newly proposed All-Payer Health Equity Approaches and Development (AHEAD) model, inviting states to adopt statewide capitated models [10–12].

Inherent in operationalizing the mandates of Maryland’s GBR was the necessity to redesign elements of care delivery which evidence shows resulted in second order effects such as centralization of complex surgical procedures and increased volume of outpatient services outside of hospital-based domains [13]. While the goal of administrators operating under various healthcare reform mandates may be assumed to be geared toward improved patient experience, the effects of their reform programs on patient experience, intended and unintended, need to be tested.

Implementation of Maryland’s GBR achieved reductions in hospital expenditures and improvements in objective measures of patient care; however, its effect(s) on patient experience, a central tenet of patient-centered orientation to healthcare delivery, remains largely unknown. The aim of this study was to evaluate the influence of Maryland’s GBR on several domains of patient’s experience of inpatient care (a direct target of the GBR in cost containment and quality terms). Patient hospital ratings are factored into the size of global budget adjustments, incenting hospitals to focus on patient experiences to improve their ratings. Thus, we hypothesized that implementation of this all-payer model would be associated with improvements in measured patient experiences with care.

## Study data and methods

### Data samples

We used the Hospital Consumer Assessment of Healthcare Providers and Systems (HCAHPS) survey from the CMS Hospital Compare database to examine patient reported experiences with inpatient care. HCAHPS is a standardized, publicly reported survey of patient perspectives of hospital care administered to a random sample of adult patients across medical conditions between 48 hours and six weeks after hospital discharge [14]. The survey evaluates patient experience across several domains including: communication with nurses and doctors, responsiveness of hospital staff, cleanliness and quietness of the hospital environment, communication about medication, discharge information, overall rating of the hospital, and if they would recommend the hospital to others. Survey response options are “never,” “sometimes,” “usually,” or “always” for all measures except for discharge information (yes/no), hospital recommendation (“definitely no,” “probably no,” “probably yes,” or “definitely yes”), and overall hospital rating (0, worst possible hospital to 10, best possible hospital). Surveys are administered through mail, email, or telephone and results are publicly available each year. Hospitals with completed HCAHPS surveys between 2010–2016 were included in this study. The full HCAHPS survey is publicly available at https://www.hcahpsonline.org/en/survey-instruments/.

Hospitals identified for inclusion from the HCAHPS database were then matched, based on county location, to area level characteristics using the Area Health Resource File (AHRF). This is an annual compilation of more than 6,000 variables collected from more than 50 databases to provide comprehensive county level information for health facilities. We used the AHRF to identify county-level health related characteristics that may influence patient-facing hospital operations including: county level number of inpatient days, number of Medicaid days, number of Medicare days, population percentage of those greater than 65 years of age, percentage of full-time registered nurses (FTE RNs), rate of preventable hospital stays, and percentage of those in poverty [15].

### Exclusion criteria

States began adoption of the Caregiver Advise, Record, Enable (CARE) act in 2014. The CARE act required hospitals to notify patients of the opportunity to designate a caregiver while also engaging and enabling the caregiver in a patient’s care by providing education on any home healthcare tasks [16]. Recent evidence suggests that passage of this act may have resulted in improved patient experiences (measured by the HCAHPS instrument) and would confound examination of the effect of the GBR [16]. The state of Maryland adopted the CARE act in 2016, hence, we excluded states that adopted the CARE act prior to 2016 from analysis [16]. Other exclusion criteria include the United States territories and states that have not expanded Medicaid or did not expand Medicaid at the time of Maryland’s Medicaid expansion in January 2014. A summary of states chosen for inclusion in the analysis based on the status of CARE passage and Medicaid expansion is shown in **S1 Table**.

### Exposure and statistical analysis

The primary outcome of this study was patient-reported experiences measured by the HCAHPS survey aggregated to the hospital for each year of the study (2010–2016). To examine the potential influence of Maryland’s GBR on patient experiences, we created a treatment indicator for the GBR. For hospitals in states without the GBR (i.e., all states other than Maryland) the treatment indicator variable was set equal to 0. For hospitals in Maryland, the variable was set equal to 0 before 2014, and equal to 1 from 2014–2016. All county level variables were log-transformed and normalized.

Statistical analyses include two-sided, two-sample t-tests for comparing county level number of inpatient days, number of Medicaid days, number of Medicare days, population percentage of those greater than 65 years of age, percentage of FTE RNs, rate of preventable hospital stays, and percentage of those in poverty between Maryland and Medicaid expanded non-GBR states. We deployed a fixed effects difference-in-difference (DID) analyses to assess differences in average treatment effect (ATT) of patient-reported experiences for GBR implemented and non-GBR implemented states, before and after implementation. Briefly, A difference-in-difference analysis is a statistical technique used to compare the changes in outcomes over time between a group that experienced an intervention and a group that did not, helping to isolate the effect of the intervention. The outcome variable for each patient-reported experience domain was defined as the percentage of “always” for nursing communication, doctor communication, help from hospital staff, pain controlled, medication communication, quietness of patient environment, and cleanliness of patient environment. Additionally, the experience domain was defined as the percentage of “yes” for discharge information, “definitely yes” for hospital recommendation, and a 9–10 rating for overall hospital rating. To test for violations of the parallel trends assumption, we trended the unadjusted responses across the study period **(S2 Table)** and used event study regression to assess that the average best-case response over time **(S1 Fig)** for the intervention group and the control group were parallel before the policy intervention. DID analyses included both univariable models and multivariable models with previously outlined county level covariates controlled by inverse probability weighting estimation. This study utilized publicly available and de-identified hospital-level and county-level data and was therefore exempt from institutional review board approval.

## Study results

### Cohort characteristics

From 2010 to 2016, a total of 11 states met the inclusion criteria (Medicaid expansion and adoption of the CARE act after 2016 or non-adoption of the CARE act) resulting in 864 hospitals eligible for inclusion in the analysis. We excluded an additional 20 hospitals (2.3% of the sample) with 0 inpatient days and stay rates of 0%. These 20 hospitals were excluded to account for noise from centers that do not have the resources to admit patients. Further, the smallest non-zero number of inpatient days and stays rates recorded for these 864 hospitals were 110 and 564, respectively. The final analytical sample of 844 hospitals includes 43 hospitals with exposure to the GBR and 801 hospitals without exposure. Hospital characteristics are displayed in **Table 1**. Overall, hospitals in Medicaid with exposure to the GBR had more inpatient days (t = 6.7, p-value < 0.05), more Medicaid days (t = 6.5, p-value < 0.05), more Medicare days (t = 6.0, p-value < 0.05), a larger FTE RN percentage (t = 5.7, p-value < 0.05), and a lower poverty percentage (t = -2.8, p-value < 0.05) across the seven year study period.

**Table 1:** Characteristics of hospitals according to GBR program status, including all years, inpatient days > 40, stays rate > 0, and non-missing data.

Hospital Characteristics	GBR Program (n = 43)	Non-GBR Program (n = 801)	t-test statistic, df, p-value
**Inpatient Days, mean (sd)**	508,087 (531,917)	286,475 (532,484)	**t = 6.7095, df = 5676, p-value = 2.143e-11**
**Medicaid Days, mean (sd)**	115,457 (135,927)	61,079 (118,436)	**t = 6.4867, df = 293.24, p-value = 3.722e-10**
**Medicare Days, mean (sd)**	199,031 (192,535)	126,524 (231,992)	**t = 6.006, df = 313.27, p-value = 5.268e-09**
**Population > 65, mean (sd)**	62,975 (44,277)	60,851 (119,245)	t = 0.67784, df = 504.39, p-value = 0.4982
**FTE RN, mean (sd)**	3,873 (4,098)	2,316 (4,417)	**t = 5.7023, df = 5676, p-value = 1.241e-08**
**Stays Rate, mean (sd)**	5,052 (1,331)	5,031 (1,978)	t = 0.24089, df = 335.82, p-value = 0.8098
**Poverty %, mean (sd)**	13.8 (7.49)	15.08 (5.61)	**t = -2.7789, df = 287.6, p-value = 0.005814**

### Difference-In-difference comparison of patient experiences

**Fig 1:**
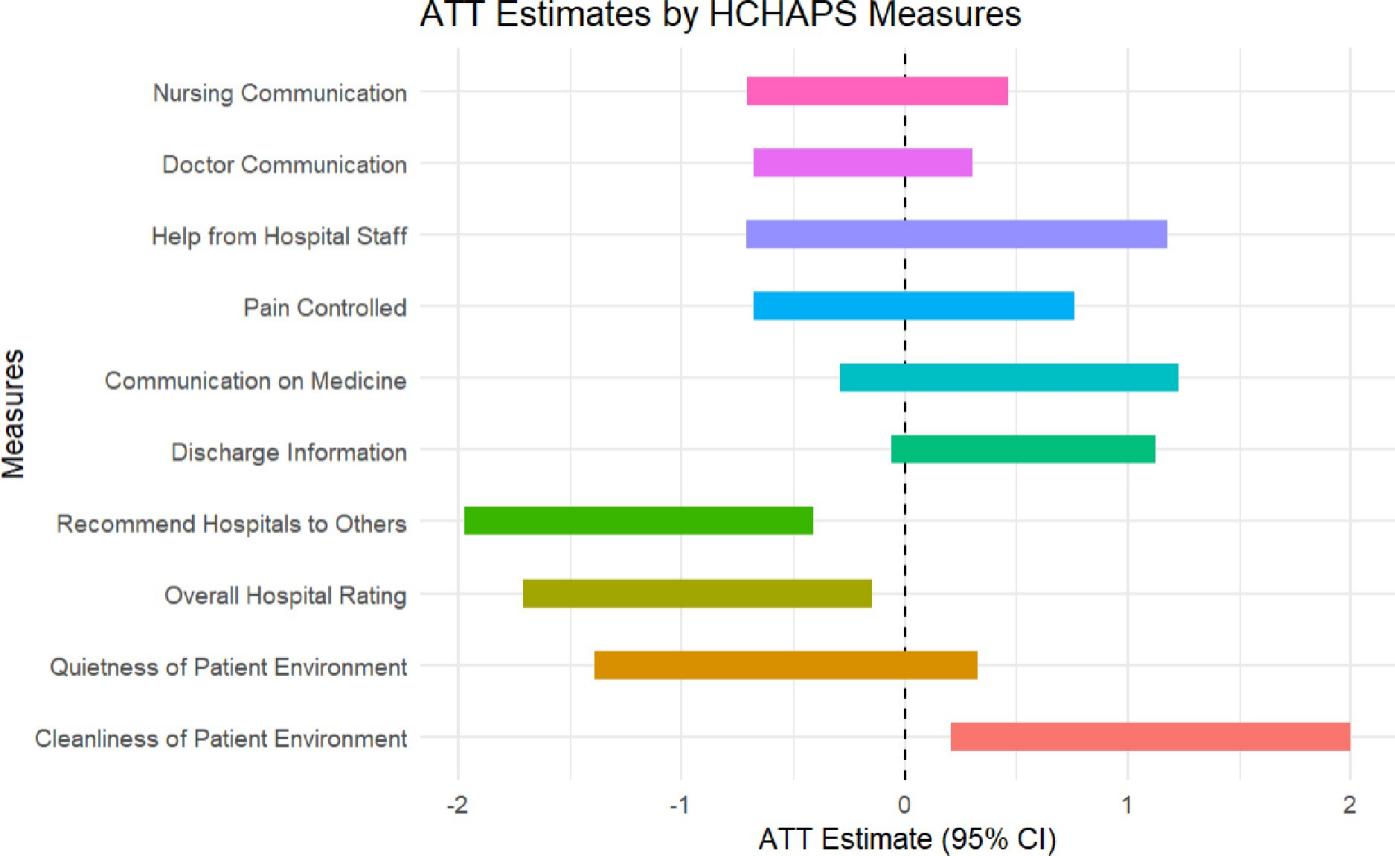
Average treatment effect (ATT) of patient-reported experiences according to measured HCAHPS domains for GBR implemented and non-implemented states.

Average treatment effect in HCAHPS domains scores in GBR and non-GBR hospitals are shown in **Fig 1** and **Table 2**. The parallel trends assumption was not violated for any HCAHPS domains. There were significant differences in HCAHPS score changes across several domains of patient experience. Compared to hospitals in non-GBR states, hospitals in GBR states experienced significant declines in the following HCAHPS domains: “would definitely recommend the hospital to others” (ATT = -1.19, 95% CI = -1.97, -0.41) and 9–10 rating of the hospital (ATT = -0.93, 95% CI = -1.71, -0.15). Results also showed significant increases in the HCAHPS domains: “if patient’s rooms and bathroom were always kept clean” (ATT = 1.10, 95% CI = 0.20, 2.00). There were no significant differences in changes for the other domains, including no improvements in: nursing communication, doctor communication, help from hospital staff, pain control, communication on medicines, discharge information, and quietness of the patient environment. The unadjusted mean HCAHPS scores according to GBR program status over the entire study period and pre/post-2014 are shown in **Table 3**.

**Table 2:** Group-time average treatment effect (ATT) in measured patient experiences according to HCAHPS domains between GBR and non-GBR program hospitals, unadjusted and adjusted with covariates.

HCAHPS patient experience domain	Unadjusted ATT (95% CI)	Adjusted ATT (95% CI)
**Nursing communication**	-0.12 (-0.7152, 0.4753)	-0.12 (-0.7048, 0.4649)
**Doctor communication**	-0.1861 (-0.6712, 0.299)	-0.1861 (-0.6768, 0.3046)
**Help from hospital staff**	-0.738 (-2.351, 0.875)	0.2326 (-0.7106, 1.1758)
**Pain controlled**	-0.1785 (-1.2557, 0.8987)	0.0415 (-0.6775, 0.7606)
**Communication on medicine**	-0.0527 (-1.355, 1.2496)	0.4693 (-0.2918, 1.2303)
**Discharge information**	0.1512 (-0.8229, 1.1252)	0.5319 (-0.0618, 1.1255)
**Recommend hospital to others**	-1.4902 (-3.6059, 0.6255)	**-1.1898 (-1.9702, -0.4094)**
**Overall hospital rating**	-1.4733 (-3.3528, 0.4061)	**-0.9289 (-1.712, -0.1457)**
**Quietness of patient environment**	-0.9359 (-2.7145, 0.8428)	-0.529 (-1.3875, 0.3295)
**Cleanliness of patient environment**	0.8878 (-0.5907, 2.3664)	**1.1015 (0.2045, 1.9985)**

**Table 3:** Characteristics of HCAHPS unadjusted mean score according to GBR program status over entire study period and pre/post-2014.

HCAHPS patient experience domain	GBR Program Unadjusted score, mean (SD), %	Non-GBR Program Unadjusted score, mean (SD), %	*p*-value
**Nursing communication** Overall Pre-2014 Post-2024	74.76 (4.93) 74.2 (4.76) 75.47 (5.06)	79.64 (5.25) 78.69 (5.1) 80.84 (5.2)	**t = 15.676, df = 4892, p-value < 2.2e-16** **t = 11.119, df = 2734, p-value < 2.2e-16** **t = 11.554, df = 2156, p-value < 2.2e-16**
**Doctor communication** Overall Pre-2014 Post-2014	77.71 (3.67) 77.48 (3.52) 77.99 (3.84)	81.36 (5.25) 80.8 (5.21) 82.07 (5.22)	**t = 16.206, df = 385.55, p-value < 2.2e-16** **t = 11.119, df = 2734, p-value < 2.2e-16** **t = 11.554, df = 2156, p-value < 2.2e-16**
**Help from hospital staff** Overall Pre-2014 Post-2014	58.63 (6.8) 58.14 (6.79) 59.25 (6.79)	68.93 (8.75) 67.91 (8.46) 70.22 (8.94)	**t = 24.956, df = 368.46, p-value < 2.2e-16** **t = 17.767, df = 202.59, p-value < 2.2e-16** **t = 17.661, df = 163.68, p-value < 2.2e-16**
**Pain controlled** Overall Pre-2014 Post-2024	66.92 (4.44) 66.77 (4.07) 67.11 (4.87)	70.92 (5.56) 70.47 (5.31) 71.49 (5.81)	**t = 14.88, df = 364.7, p-value < 2.2e-16** **t = 11.166, df = 206.15, p-value < 2.2e-16** **t = 9.9178, df = 157.77, p-value < 2.2e-16**
**Communication on medicine** Overall Pre-2014 Post-2014	58.8 (5.11) 58.02 (4.88) 59.79 (5.24)	64.38 (6.5) 63.47 (6.21) 65.54 (6.67)	**t = 17.999, df = 366.73, p-value < 2.2e-16** **t = 13.753, df = 204.06, p-value < 2.2e-16** **t = 12.026, df = 161.56, p-value < 2.2e-16**
**Discharge information** Overall Pre-2014 Post-2014	84.17 (4.12) 82.85 (4.15) 85.83 (3.43)	86.21 (4.58) 85.06 (4.43) 87.66 (4.34)	**t = 8.255, df = 350.37, p-value = 3.133e-15** **t = 6.2813, df = 2733, p-value = 3.893e-10** **t = 5.849, df = 161.11, p-value = 2.677e-08**
**Recommend hospital to others** Overall Pre-2014 Post-2014	66.19 (8.79) 66.48 (8.49) 65.82 (9.17)	71.86 (9.56) 71.37 (9.49) 72.49 (9.61)	**t = 10.032, df = 4892, p-value < 2.2e-16** **t = 6.5164, df = 2734, p-value = 8.545e-11** **t = 7.7747, df = 2156, p-value = 1.162e-14**
**Overall hospital rating** Overall Pre-2014 Post-2014	64.55 (7.68) 64.18 (7.41) 65.03 (8.02)	71.52 (8.52) 70.41 (8.31) 72.93 (8.58)	**t = 15.135, df = 350.17, p-value < 2.2e-16** **t = 9.4786, df = 2734, p-value < 2.2e-16** **t = 10.326, df = 2156, p-value < 2.2e-16**
**Quietness of patient environment** Overall Pre-2014 Post-2014	56.29 (5.89) 55.64 (5.06) 57.11 (6.73)	60.17 (9.6) 58.88 (9.48) 61.82 (9.5)	**t = 10.554, df = 412.68, p-value < 2.2e-16** **t = 7.4659, df = 251.6, p-value = 1.353e-12** **t = 7.5958, df = 168.65, p-value = 2.004e-12**
**Cleanliness of patient environment** Overall Pre-2014 Post-2014	65.29 (5.73) 64.71 (5.82) 66.02 (5.55)	74.64 (7.94) 74.18 (7.66) 75.23 (8.25)	**t = 26.699, df = 379.91, p-value < 2.2e-16** **t = 19.995, df = 206.8, p-value < 2.2e-16** **t = 17.889, df = 172.9, p-value < 2.2e-16**

## Discussion

In this study examining the effect of Maryland’s GBR on patient experiences with inpatient care, we found that the GBR program was associated with a significant increase in the HCAHPS domains “if patient’s rooms and bathroom were always kept clean”, but also significant decreases in some domains of patient experience. Following adoption of the GBR, a lower percentage of patients discharged from hospitals operating under the GBR would recommend the hospitals to family and friends and a lower percentage of patients would give the hospitals the highest rating compared to patients in non-GBR states. These findings are pertinent in the context of accelerating trends in healthcare delivery and payment reform efforts because they show potential “spill-over” effects that may adversely impact important planks of delivery reform like patient-centeredness.

Evaluation of the Comprehensive Care for Joint Replacement program demonstrated lowered expenditures largely from less discharges to post-acute care facilities with spillover effects of similar size and magnitude in post-acute care related expenditures in non-targeted populations [17]. Offodile et al. in their recent study, theorized that implementation of the GBR model may incent hospitals in aggregate to allocate resources to cost-efficient and high-quality services resulting in an unintended centralization of care for complex patients. They demonstrated that following implementation of the GBR model, some complex surgical procedures including gastrectomy, pneumonectomy/lobectomy, and hip/knee revision procedures were increasingly performed at high-concentration hospitals in Maryland [13]. The effect of this centralization of complex services on access and ancillary burden on patients and caregivers, which can influence care experiences is unknown. Therefore, with the acknowledgement that full risk capitated models have so far been superior in achieving cost-containment goals compared to other payment reforms [18, 19], we must heed the cautionary dictum that whole population oriented capitated models like Maryland’s GBR are effective but may also be blunt instruments for which additional considerations must be taken to mitigate adverse unintended consequences.

It is unclear from this study why there were declines in the percentages of top hospital ratings and recommendation of hospitals to loved ones. Other study designs and data sources would shed light on this question; however, our study results are not isolated in their substance. Recent external evaluations of the GBR and subsequent TCOC model, also found that Maryland hospitals were rated lower than comparison groups on nearly all measures of patient experience, including patient hospital ratings, despite a reported focus by hospital leaders to improve patient experiences [1, 2, 12]. Together, these findings demonstrate the need for focused attention on the broader impact that redesign strategies have on patient’s experiences with care. Although data from the HCAHPS survey do surmise patient sentiment on broad themes of care experience, they lack granularity to offer specific insights for problem solving and lack in timeliness for prompt resolution as there is a lag between episodes of care, completion of surveys, and availability of results. These factors may contribute to putting providers on the backfoot in efforts to improve patient ratings of their care experience. A recent systematic review examining strategies to improve hospital care satisfaction based on HCAHPS domains concluded that the peer-reviewed record of interventions lacks comprehensiveness and leaves administrators with little more than anecdotes to guide strategic decision making [20]. Grievance and complaint resolution mechanisms are statutorily mandated as a component of Medicare Conditions of Participation. Arguably, these mechanisms can provide more granular and timely details about issues that influence ratings as they originate expressively from patients, families, and caregivers proximate to the episode of care in question. Additionally, given the free form and open nature of the process, details regarding wider domains of care experience than that provided by HCAHPS can be garnered. Grievance and complaint mechanisms should remain a plank of assessing patient experience with care delivery especially with efforts researchers have taken to taxonomize complaints for more efficacious resolution [21, 22]. However, with a specific focus on unintended adverse effects of planned care transformation initiatives, preemptive efforts should be taken to herd off potential adverse effects.

Although factoring patient hospital ratings into the size of global budget adjustments, as Maryland’s model does, incents hospitals to focus on patient experiences to improve their ratings, this focus should come with insights into pertinent patient needs. An example of this can be found in another statewide policy initiative which offers a precedent for how pertinent patient needs are considered in shaping reform priorities. The state of Oregon’s Coordinated Care Organizations (CCOs), established by the state’s legislature following passage of the Affordable Care Act, are local networks of providers who provide care to Medicaid enrollees under a capitated funding mechanism. By law, CCOs are required to have at least one Community Advisory Council (CAC) that among other functions help guide organizational priorities with health needs assessments and community specific plans for impactful services and improvements in care delivery. Although, to date, there has not been an assessment of the impact of Oregon’s CACs on the overarching objectives of cost containment and improvement of healthcare access and quality nor on patient experience of care which is the focus of this study, on the face of it, there is merit to provider engagement with communities on reform initiatives which may in intended and unintended ways impact their experience with care. The CARE act is an example of improved experience of care through response to community priorities in care delivery. The CARE act is the product of advocacy on behalf of the community served by AARP that explicitly puts on providers the burden of communication with designated caregivers to enhance care transitions following hospitalization. In a comprehensive comparison of hospitals in states that adopted the CARE act to those in states that did not, researchers found differential improvements in patient ratings for nurse and physician communication, provision of discharge information, and hospital ratings in hospitals under the CARE act mandate [16]. With emphasis on collaboration with caregivers during the discharge process, patients reported improved preparedness for discharge and improved communication resulting in measurable improvements in patient care experience [16]. So far, the CARE act has been adopted in 42 states including the state of Maryland in 2016. Hence, it warrants examination if adoption of the patient-centered CARE act in Maryland reverses the negative trend in patient ratings that this study found following GBR implementation.

### Study limitations

There are several limitations that should be noted in this study. The focus of this study was to analyze patient experiences with care according to measured HCAHPS domains; however, these measures are not entirely representative of the quality of a patient’s care and their experiences with care and ultimately limits the generalizability of the findings of this study. Despite this, HCAHPS scores are a valid assessment of hospital quality, and a metric that is used in quality reporting and value-based purchasing programs [12]. As this was an observational study, there may be unobserved factors responsible for the differential changes seen, despite attempting to adequately control for competing factors and hospital characteristics. Furthermore, the hospital overall star rating was not implemented until July 2016, and we were unable to incorporate this measure in our analysis. Lastly, our analysis terminated at the end of 2016 due to the implementation of the CARE program. Therefore, the findings of our study may reflect early effects and experiences of the GBR model.

## Conclusion

The results of this study suggest that implementation of a full risk capitated hospital funding model in the state of Maryland was associated with differential decreases in HCAHPS patient experience scores, specifically with decreases in overall hospital rating and recommending the hospital to others, compared to hospitals in states without similar funding mechanisms. These findings suggest there should be efforts made to ascertain and mitigate potential adverse effects of care transformation initiatives on patient experience. Patients are stakeholders and their inputs should be sought and incorporated in care transformation efforts to ensure that these cost-saving and outcomes improving models align with improved patient experiences.

## Supporting information

S1 Fig Average best-case response percentage over time by intervention groups. Solid line represents a simple linear regression before and after implementation. Grey shading represents 95% CI.
(DOCX)

S1 Table Showing a panel of figures according to the measured HCHAPS domains and differential change in cases vs. controls. (DOCX)

S2 Table Event study regression results in measured patient experiences according to HCHAPS domains between GBR and non-GBR program hospitals across all years. (DOCX)

## References

[1] Susan Haber MM, Leslie Greenwald, Rebecca Perry, Linda Jiang, Sam Masters, Regina Rutledge, et al. EVALUATION OF THE MARYLAND ALL-PAYER MODEL VOLUME II: FINAL REPORT APPENDICES 2019.

[2] Services CfMM. EVALUATION OF THE MARYLAND ALL-PAYER MODEL VOLUME I: FINAL REPORT. 2019.

[3] Aliu O, Lee AWP, Efron JE, Higgins RSD, Butler CE, Offodile AC II. Assessment of Costs and Care Quality Associated With Major Surgical Procedures After Implementation of Maryland’s Capitated Budget Model. JAMA Network Open. 2021;4(9):e2126619-e. doi: 10.1001/jamanetworkopen.2021.26619 34559228 PMC8463941

[4] Shammas RL, Coroneos CJ, Ortiz-Babilonia C, Graton M, Jain A, Offodile ACI. Implementation of the Maryland Global Budget Revenue Model and Variation in the Expenditures and Outcomes of Surgical Care: A Systematic Review and Meta-Analysis. Annals of Surgery. 9900:10.1097/SLA.0000000000005744. doi: 10.1097/SLA.0000000000005744 -990000000-00301. 36314127

[5] Haber S, Beil H, Morrison M, Greenwald L, Perry R, Jiang L, et al. Evaluation of the Maryland All-Payer Model, Volume I: Final Report. Waltham, MA: RTI International. 2019.

[6] Beil H, Haber SG, Giuriceo K, Amico P, Morrison M, Beadles C, et al. Maryland’s Global Hospital Budgets: Impacts on Medicare Cost and Utilization for the First 3 Years. Med Care. 2019;57(6):417–24. doi: 10.1097/MLR.0000000000001118 . 30994523

[7] Papanicolas I, Woskie LR, Jha AK. Health Care Spending in the United States and Other High-Income Countries. Jama. 2018;319(10):1024–39. doi: 10.1001/jama.2018.1150 . 29536101

[8] Muñoz E, Muñoz W 3rd, Wise L. National and surgical health care expenditures, 2005–2025. Ann Surg. 2010;251(2):195–200. doi: 10.1097/SLA.0b013e3181cbcc9a . 20054269

[9] Burwell SM. Setting value-based payment goals—HHS efforts to improve U.S. health care. N Engl J Med. 2015;372(10):897–9. Epub 20150126. doi: 10.1056/NEJMp1500445 . 25622024

[10] Scanlon D, Sciegaj M, Wolf LJ, Vanderbrink J, Johannes B, Shaw B, et al. The Pennsylvania Rural Health Model: Hospitals’ Early Experiences With Global Payment for Rural Communities. J Healthc Manag. 2022;67(3):162–72. Epub 20220501. doi: 10.1097/JHM-D-20-00347 . 35261348

[11] Three Outstanding Questions About CMS’s Ambitious New AHEAD Model 2023 [updated September 14].

[12] Jason Rotter KC, Kate Stewart, Isabel Platt, Rachel Machta, Keith Kranker, Nancy McCall, Greg Peterson. Evaluation of the Maryland Total Cost of Care Model: Quantitative-Only Report for the Model’s First Three Years (2019 to 2021). https://www.cms.gov/priorities/innovation/data-and-reports/2022/md-tcoc-qor2. 2022.

[13] Offodile AC 2nd, Lin YL, Shah SA, Swisher SG, Jain A, Butler CE, et al. Is the Centralization of Complex Surgical Procedures an Unintended Spillover Effect of Global Capitation?—Insights from the Maryland Global Budget Revenue Program. Ann Surg. 2023;277(4):535–41. Epub 20221027. doi: 10.1097/SLA.0000000000005737 ; PubMed Central PMCID: PMC9994796. 36512741 PMC9994796

[14] Centers for Medicare & Medicaid Services. HCAHPS: Patients’ Perspectives of Care Survey cms.gov2023. Available from: https://www.cms.gov/medicare/quality/initiatives/hospital-quality-initiative/hcahps-patients-perspectives-care-survey.

[15] Area Health Resources Files. 2023. https://data.hrsa.gov/topics/health-workforce/ahrf.

[16] Lee CR, Taggert E, Coe NB, Chatterjee P. Patient Experience at US Hospitals Following the Caregiver Advise, Record, Enable (CARE) Act. JAMA Network Open. 2023;6(5):e2311253-e. doi: 10.1001/jamanetworkopen.2023.11253 37126344 PMC10152302

[17] Einav L, Finkelstein A, Ji Y, Mahoney N. Randomized trial shows healthcare payment reform has equal-sized spillover effects on patients not targeted by reform. Proc Natl Acad Sci U S A. 2020;117(32):18939–47. Epub 20200727. doi: 10.1073/pnas.2004759117 ; PubMed Central PMCID: PMC7431052. 32719129 PMC7431052

[18] Offodile AC 2nd, Gibbons JB, Murrell S, Kinzer D, Sharfstein JM. A Global Equity Model (GEM) for the Advancement of Community Health and Health Equity. NAM Perspect. 2022;2022. Epub 20221114. doi: 10.31478/202211b ; PubMed Central PMCID: PMC9875856. 36713771 PMC9875856

[19] Kadakia KT, Offodile AC 2nd. The Next Generation of Payment Reforms for Population Health—An Actionable Agenda for 2035 Informed by Past Gains and Ongoing Lessons. Milbank Q. 2023;101(S1):866–92. doi: 10.1111/1468-0009.12632 ; PubMed Central PMCID: PMC10126963. 37096610 PMC10126963

[20] Davidson KW, Shaffer J, Ye S, Falzon L, Emeruwa IO, Sundquist K, et al. Interventions to improve hospital patient satisfaction with healthcare providers and systems: a systematic review. BMJ Qual Saf. 2017;26(7):596–606. Epub 20160803. doi: 10.1136/bmjqs-2015-004758 ; PubMed Central PMCID: PMC5290224. 27488124 PMC5290224

[21] Reader TW, Gillespie A, Roberts J. Patient complaints in healthcare systems: a systematic review and coding taxonomy. BMJ Qual Saf. 2014;23(8):678–89. Epub 20140529. doi: 10.1136/bmjqs-2013-002437 ; PubMed Central PMCID: PMC4112446. 24876289 PMC4112446

[22] Dael Jv, Reader TW, Gillespie A, Neves AL, Darzi A, Mayer EK. Learning from complaints in healthcare: a realist review of academic literature, policy evidence and front-line insights. BMJ Quality & Safety. 2020;29(8):684–95. doi: 10.1136/bmjqs-2019-009704 32019824 PMC7398301

