# Supplementary material for: Patient experience with hospital care following the Maryland global budget revenue model: A difference-in-difference analysis
Source: PLoS One. 2024 Aug 6;19(8):e0308331. doi: 10.1371/journal.pone.0308331 (PMC11302862; doi:10.1371/journal.pone.0308331)
Supplement: S1 Fig — Solid line represents a simple linear regression before and after implementation. Grey shading represents 95% CI. (DOCX) [file pone.0308331.s001.docx]

| **S1 Figure:** Average best-case response percentage over time by intervention groups. Solid line represents a simple linear regression before and after implementation. Grey shading represents 95% CI. |
| --- |
| 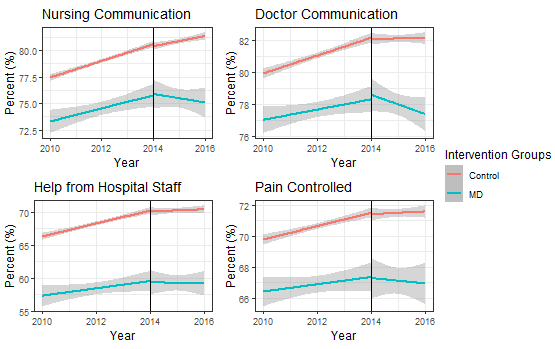  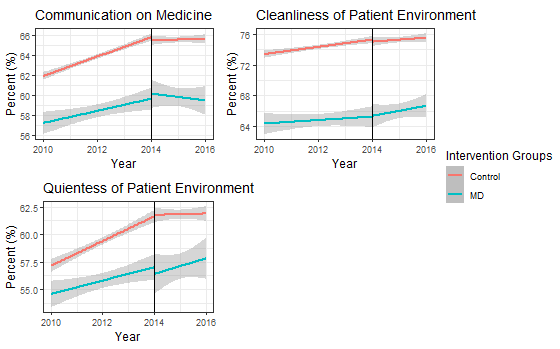  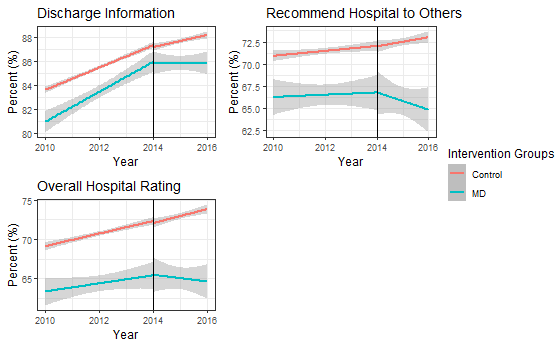 |
