# Supplementary material for: Patient experience with hospital care following the Maryland global budget revenue model: A difference-in-difference analysis
Source: PLoS One. 2024 Aug 6;19(8):e0308331. doi: 10.1371/journal.pone.0308331 (PMC11302862; doi:10.1371/journal.pone.0308331)
Supplement: S1 Table — (DOCX) [file pone.0308331.s002.docx]

**S1 Table:** Showing a panel of figures according to the measured HCHAPS domains and differential change in cases vs. controls.

| **GBR Program States** | **Non-GBR Program States** | **Medicaid Expansion States** |
| --- | --- | --- |
| MD | AK, AL, AZ, DE, FL, GA, HI, IA, ID, KS, KY, MA, MN, MO, MT, NC, ND, OH, PA, RI, SC, SD, TN, TX, VT, WI | RI, OH, MT, MN, MA, KY, HI, DE, ND, IA, AZ |
