# Supplementary material for: Patient experience with hospital care following the Maryland global budget revenue model: A difference-in-difference analysis
Source: PLoS One. 2024 Aug 6;19(8):e0308331. doi: 10.1371/journal.pone.0308331 (PMC11302862; doi:10.1371/journal.pone.0308331)
Supplement: S2 Table — (DOCX) [file pone.0308331.s003.docx]

| **S2 Table:** Event study regression results in measured patient experiences according to HCHAPS domains between GBR and non-GBR program hospitals across all years. | | |
| --- | --- | --- |
| **HCHAPS patient experience domain** | **ATT (95% CI)** | **Parallel Trends *p*-value** |
| **Nursing communication**  **2011**  **2012**  **2013**  **2014**  **2015**  **2016** | -0.1419 (-2.6299, 2.3461)  0.4210 (-2.0996, 2.9416)  -1.3365 (-4.1751,1.5020)  0.7925 (-1.5552, 3.1401)  -0.1076 (-2.8718, 2.6565  -0.6907 (-3.4852, 2.1039) | 0.54305 |
| **Doctor communication**  **2011**  **2012**  **2013**  **2014**  **2015**  **2016** | 0.3034 (-1.9201, 2.5269)  -0.3423 (-2.4691, 1.7845)  -0.9386 (-2.8137, 0.9365)  0.5300 (-1.4107, 2.4706)  0.0339 (-2.1744, 2.2423)  -0.7938 (-2.8633, 1.2756) | 0.30385 |
| **Help from hospital staff**  **2011**  **2012**  **2013**  **2014**  **2015**  **2016** | 0.0743 (-3.7500, 3.8986)  0.3532 (-3.4463, 4.1528)  -2.6585 (-6.6355, 1.3184)  1.3487 (-2.4552, 5.1526)  -0.3133 (-4.4527, 3.8260)  1.4864 (-2.0811, 5.0538) | 0.29508 |
| **Pain controlled**  **2011**  **2012**  **2013**  **2014**  **2015**  **2016** | 0.4975 (-2.0357, 3.0307)  0.1826 (-1.8978, 2.2630)  -1.4266 (-3.6703, 0.8170)  0.5218 (-1.7015, 2.7451)  -0.2366 (-3.3980, 2.9249)  0.3158 (-2.3954, 3.0270) | 0.39862 |
| **Communication on medicine**  **2011**  **2012**  **2013**  **2014**  **2015**  **2016** | -0.5214 (-3.3960, 2.3531)  0.2121 (-2.5477, 2.9719)  -1.9126 (-4.7425, 0.9172)  1.1765 (-1.3652, 3.7181)  1.7282 (-1.4010, 4.8575)  0.4856 (-2.2339, 3.2051) | 0.12086 |
| **Discharge information**  **2011**  **2012**  **2013**  **2014**  **2015**  **2016** | 0.5395 (-1.8124, 2.8913)  0.4012 (-1.9774, 2.7798)  0.0863 (-1.9559, 2.1285)  0.2342 (-1.6902, 2.1586)  -0.1879 (-2.0283, 1.6526)  -0.6442 (-2.6911, 1.4026) | 0.60301 |
| **Recommend hospital to others**  **2011**  **2012**  **2013**  **2014**  **2015**  **2016** | 0.7799 (-3.2345, 4.7943)  -0.2219 (-4.8344, 4.3906)  -0.8043 (-5.3483, 3.7397)  0.2159 (-4.2730, 4.7048)  -1.2352 (-6.0544, 3.5841)  -2.5482 (-7.5669, 2.4705) | 0.93134 |
| **Overall hospital rating**  **2011**  **2012**  **2013**  **2014**  **2015**  **2016** | 0.9264 (-2.8320, 4.6848)  -0.7323 (-4.8034, 3.3387)  -0.5019 (-4.4048, 3.4010)  0.1471 (-3.8970, 4.1913)  -0.6249 (-4.7536, 3.5037)  -2.3872 (-6.8165, 2.0420) | 0.86714 |
| **Quietness of patient environment**  **2011**  **2012**  **2013**  **2014**  **2015**  **2016** | 0.3923 (-2.9288, 3.7134)  -0.4012 (-3.5012, 2.6988)  -0.6690 (-3.9012, 2.5632)  -0.8069 (-4.0037, 2.3898)  -0.9275 (-4.8556, 3.0005)  0.4273 (-3.8201, 4.6747) | 0.86926 |
| **Cleanliness of patient environment**  **2011**  **2012**  **2013**  **2014**  **2015**  **2016** | 0.9636 (-2.7681, 4.6953)  -0.3517 (-3.9216, 3.2181)  -1.5330 (-4.9271, 1.8611)  1.3702 (-1.8945, 4.6350)  1.5583 (-1.5986, 4.7153)  2.2926 (-0.9336, 5.5188) | 0.54546 |
